# The injury progression of T lymphocytes in a mouse model with subcutaneous injection of a high dose of sulfur mustard

**DOI:** 10.1186/s40779-014-0028-8

**Published:** 2014-12-19

**Authors:** Yi-Zhou Mei, Xiao-Rui Zhang, Ning Jiang, Jun-Ping Cheng, Feng Liu, Pan Zheng, Wen-Xia Zhou, Yong-Xiang Zhang

**Affiliations:** State Key Laboratory of Toxicology and Medical Countermeasures, Beijing Institute of Pharmacology and Toxicology, Beijing, 100850 China

**Keywords:** Sulfur mustard, T lymphocyte, Apoptosis, Cytokine, DNA damage

## Abstract

**Background:**

In clinical studies, the findings on sulfur mustard (SM) toxicity for CD3^+^CD4^+^ and CD3^+^CD8^+^ T lymphocyte subsets are contradictory. In animal experiments, the effect of SM on the T cell number and proliferation is incompatible and is even the opposite of the results in human studies. In this study, we observed the dynamic changes of T lymphocytes in the first week in a high-dose SM-induced model.

**Methods:**

Mice were exposed to SM by subcutaneous injection (20 mg/kg) and were sacrificed 4 h, 24 h, 72 h and 168 h later. Spleen T lymphocyte proliferation was evaluated by ^3^H-TdR. Flow cytometric analysis was used to observe the percentage of CD3^+^CD4^+^ and CD3^+^CD8^+^ T lymphocyte subsets. The IL-1β, IL-6, IL-10 and TNF-α levels in plasma were assayed using the Luminex method. DNA damage in bone marrow cells was observed with the single cell gel electrophoresis technique (SCGE).

**Results:**

SM continuously inhibited the proliferation of lymphocytes for 7 days, and there was a significant rebound of Con A-induced T lymphocyte proliferation only at 24 h. The percentage of CD3^+^CD4^+^ and CD3^+^CD8^+^ lymphocytes was upregulated, which was accompanied by increased IL-1β and TNF-α and decreased IL-10. The IL-6 level was gradually decreased in the PG group at 4 h. The peak of lymphocytic apoptosis and DNA damage occurred at 24 h and 72 h, respectively.

**Conclusion:**

Our results show that SM significantly inhibited T lymphocyte proliferation as well as induced CD3^+^CD4^+^ and CD3^+^CD8^+^ upregulation. SM intoxication also significantly increased the levels of pro-inflammatory cytokines (IL-1β, IL-6 and TNF-α) and inhibited the level of anti-inflammatory cytokine IL-10. Our results may partly be due to the significant SM induced significant apoptosis and necrosis of lymphocytes as well as DNA damage of bone marrow cells. The results provided a favorable evaluation of SM immune toxicity in an animal model.

## Background

Sulfur mustard (bis(2-chloroethyl) sulfide, sulfur mustard; SM), a highly reactive alkylating agent, was used as a chemical warfare agent in World War I/II and the Iraq/Iran conflict. SM can inflict damage in multiple organs, especially the skin and eyes, as well as the respiratory tract, via complex mechanisms [1-8]. SM can be absorbed into the bloodstream through the skin or digestive tract, thus leading to the damage of multiple systems, such as the immune system, and causing subsequent complex changes [9,10]. A detailed immunological consequence of SM exposure of Iran victims in the Iraq-Iran war has been reported [11]. The T cell and monocyte counts in patients exposed to SM reduced to less than 54% and 65% of normal within one week after exposure, respectively. However, some studies found that T lymphocytes were relatively unaffected compared with B lymphocytes after SM exposure in an animal model, which was not consistent with human studies [12]. The proliferation of the remaining T cells in the presence of Concanavalin A or to an anti-CD3 antibody was not significantly affected [12]. Due to the importance of T lymphocytes in SM-induced injury and the contradictory results regarding T lymphocytes, further investigation was required.

Research in humans showed that, 20 years after the SM exposure, the severely affected victims of the Iran-Iraq war had a lower CD3^+^CD4^+^ level and higher CD3^+^CD8^+^ level in plasma compared to normal controls [13]. Another study in humans, found that 15 years after sulfur mustard gas exposure, participants had Sezary syndrome and increased CD3^+^CD4^+^/CD3^+^CD8^+^ cells in flow cytometry [14]. Other studies observed increased percentages of CD3^+^CD4^+^ and CD3^+^CD8^+^ T cells in tissues and body fluids in hairless guinea pigs [15]. The contradictions between human and animal models may be due to the different degrees of injury. The patients in human studies had severe SM exposure, while the animals were exposed to a low dose of SM. In animal experiments, different types of animals, SM doses, exposure routes, and observation times were used. These differences would cause inconsistent conclusions about the effect of SM on T lymphocyte proliferation and CD3^+^CD4^+^ and CD3^+^CD8^+^ T subsets. Because of the rapid recovery ability of the immune system in mice, we supposed that a higher SM dose is more suitable for developing a mouse model that imitates the toxic process in humans. Therefore, we constructed a high dose SM (20 mg/kg) exposure mouse model and observed the dynamic state of T lymphocyte function in this research. Additionally, the homeostasis of proinflammatory and anti-inflammatory cytokines in lymphocytes, apoptosis, necrosis, and DNA damage of bone marrow cells were studied, which would help elucidate the process of SM-induced immune system damage [16-18].

In this research, we developed an SM-induced mouse model by subcutaneous injection of a high dose of SM (20 mg/kg). The dynamic changes of the T lymphocytes in the model were observed at 4 h, 24 h, 72 h and 7 d after SM exposure. This study provides valuable information that could enhance our understanding of the progression of SM toxicity on immune function.

## Methods

### Materials

SM was synthesized by Beijing Institute of Pharmacology and Toxicology and formulated into the desired concentration with 1,2-propanediol solution (Sigma, St. Louis, MO, USA) before use. ^3^H-TdR was obtained from the China Institute of Atomic Energy (Beijing, China). RPMI 1640 and calf serum were from Gibco (Carlsbad, CA, USA). APC-anti-CD3, PE-anti-CD4, and FITC-anti-CD8 were from BioLegend (San Diego CA, USA). The Luminex mouse cytokine kit was from Millipore (San Diego, CA, USA). The Annexin V-FITC apoptosis kit was from Becton, Dickinson and Company (Franklin Lakes, NJ, USA). All other chemicals were obtained from Sigma (St. Louis, MO, USA) unless otherwise indicated.

### Animals

Kunming mice of clean grade, male, weighing 18–22 g, were purchased from the Experimental Animal Center of Beijing Institute of Pharmacology and Toxicology. The animals were acclimatized for 3 days before their use in our experimental work, which was performed according to a specified protocol approved by the Institutional Animal Care and Use Committee (IACUC) of Beijing Institute of Pharmacology and Toxicology. All mice were housed at room temperature (22 ± 2°C), 45%-55% relative humidity, and a 12/12 h light–dark cycle with free access to standard rodent chow and water.

### Mouse model induced by subcutaneous injection of SM

One hundred and sixty Kunming mice were randomly divided into the following three groups: control group, propylene glycol (PG) group and SM exposure group. Animals in the control group, PG group, and SM group received a subcutaneous injection of saline, PG solution, and SM (20 mg/kg), respectively. The subcutaneous injection volume was 0.1 ml/20 g in all animals, which was continuously observed for 7 days (168 h). Twelve animals in each group were sacrificed 4 h, 24 h, 72 h and 168 h after exposure.

### Lymphocyte proliferation assay

Mouse lymphocyte proliferation was observed with the ^3^H-thymidine incorporation method [19]. Six Kunming mice per group were sacrificed. Their spleens were aseptically removed and minced through a 40-μm nylon cell strainer to achieve a single-cell suspension. The red blood cells were depleted with Tris–NH_4_Cl lysis buffer (0.144 mol/L NH_4_Cl, 0.017 mol/L Tris–HCl). A total of 5 × 10^5^ spleen cells was stimulated with Con A (0.5 μg/ml). The spleen cells were cultured in RPMI 1640 medium supplemented with 10% FBS, penicillin (100 U/ml), and streptomycin (100 μg/ml) at 37°C in a 5% CO_2_ humidified incubator for 72 h, and they were pulsed with ^3^H-thymidine (1 μCi/well) during the last 18 h of incubation. The cells were harvested on glass fiber filters using a Filtermate cell harvester (Packard). The level of ^3^H-thymidine incorporated into the cells was measured with a β-scintillation counter (Beckman LS6500). The results were expressed as cpm (counters per minute) of the stimulated cells and cpm of the unstimulated cells.

### Flow cytometric analysis of CD3^+^CD4^+^ and CD3^+^CD8^+^ T lymphocytes

The lymphocytes were washed with cold PBS containing 0.1% NaN_3_ and 1% FBS. A total of 10^6^ cells were incubated with APC-conjugated anti-mouse CD3, PE-conjugated anti-mouse CD4 and FITC-conjugated anti-mouse CD8 (BD Biosciences) for 20 min at room temperature. The cells were washed in PBS containing 0.1% FBS and analyzed by flow cytometry (BD Calibur™).

### Plasma cytokine assay by Luminex^™^ 200

Blood samples from 6 animals in each group were centrifuged (4°C, 3,000 *g* × 10 min) and then stored at −70°C. The levels of IL-1β, IL-6, IL-10 and TNF-α were analyzed with a cytokine/chemokine kit (MPXMCYTO-70 K-09, Millipore Corporation).

### Flow cytometric analysis of apoptosis and necrosis

To detect phosphatidylserine, annexin V-FITC was used in a combination with propidium iodide (PI) and an ApopNexin FITC Apoptosis Detection Kit (Franklin Lakes, NJ, USA). Every flow tube contained a 0.1 ml cell suspension, and the cells were washed twice with 0.5 ml PBS solution (1,000 r/min × 5 min). The supernatant was discarded, and then 0.4 ml of binding buffer cell suspension and 5 ml of Annexin-V were added. After vortex mixing, the tubes were incubated for 15 min at 4°C in the dark. The FITC-Annexin V fluorescence was read with the FL1 photomultiplier tube, and the PI fluorescence was detected using the FL3 channel.

### Single cell gel electrophoresis technique (SCGE)

The DNA damage of bone marrow cells was evaluated by SCGE according to a previous report [20,21]. Briefly, the cells were embedded in agarose and layered on a microscope slide; they were then immersed for 1 h at 4°C in a freshly prepared lysing solution (2.5 mol/L NaCl, 100 mmol/L Na_2_-EDTA, and 10 mmol/L Tris, pH 10) and supplemented immediately prior to use with 1% N-lauroylsarcosine, 10% DMSO and 1% Triton X-100. Following the steps of alkaline unwinding (pH > 13) for 40 min, electrophoresis and neutralization were performed as a standard protocol. All steps were conducted under a dimmed light to prevent additional DNA damage. Following the electrophoresis run, the slides were neutralized and dipped into cold 100% ethanol. Then, the slides were dried at room temperature before analysis. Fifty cells from each of the duplicate slides were randomly examined under a fluorescence microscope, and the extent of DNA damage was measured using Image-Pro Plus 6.0 (IPP6.0), which performed a software-based analysis of electronic images. DNA damage was quantified as the percentage of comets and the average tail length (TL). The median of each parameter was used as a representative value for each subject, and the mean of the medians was used for statistical analysis.

$$ \begin{array}{l}\mathrm{The}\ \mathrm{formula}\ \mathrm{is}:\ \mathrm{TL} = \left(\Sigma\ \mathrm{each}\ \mathrm{cell}\ \mathrm{diameter}/\mathrm{total}\ \mathrm{cell}\mathrm{s}\right) \pm \mathrm{S}\mathrm{D}\hfill \\ {}\mathrm{Percentage}\ \mathrm{of}\ \mathrm{comets} = \left(\mathrm{comet}\ \mathrm{cell}\mathrm{s}/\mathrm{measurement}\ \mathrm{cell}\ \mathrm{number}\right) \times 100\%\hfill \end{array} $$

### Statistical analysis

The data were analyzed by GraphPad Prism Software 5.01 and SPSS Software 13.0 using Student’s *t*-test. Data were expressed as the mean ± SD and analyzed using one-way ANOVA, which was followed by the Bonferroni *t*-test for multiple comparisons. *P* < 0.05 was considered statistically significant.

## Results

### Spleen weight and coefficient

**Figure 1:**
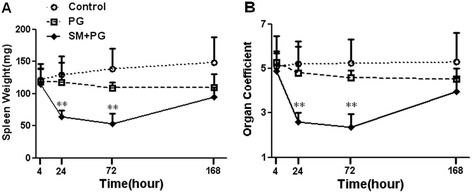
**Dynamic decrease in the spleen weight and organ coefficient of the spleen in mice exposed to sulfur mustard (SM).** Male KM mice were exposed to saline, propylene glycol (PG) and 20 mg/kg SM by subcutaneous injection. Six animals from each group were sacrificed after SM exposure at 4 h, 24 h, 72 h and 168 h, and the spleens were quickly weighed. The spleen weight **(A)** and organ coefficient of the spleen **(B)** of every animal were observed to assess SM-induced immune organ injury. Data were presented as the mean ± SD, *n* = 6 in each group **(A**-**B)**. *Significant difference between the PG treatment and SM exposure groups (*P < 0.05, **P < 0.01, ***P < 0.001).

To study the toxicity of SM on immune organs, the spleen weight and coefficient were observed. After subcutaneous injection with SM-PG solution (20 mg/kg), 6 animals were sacrificed at 4 h, 24 h, 72 h, and 168 h after SM exposure. There was no significant difference in the spleen weight or coefficient between the SM group and solvent treatment group (PG group) at 4 h. Significant decreases in the spleen weight and coefficient were observed in the SM group at 24 h and 72 h after SM exposure (*P* < 0.01). The results of the weight and coefficient of the spleen are shown in Figure 1A and B. At 168 h, the weight and coefficient of the spleen in the SM group were restored to the normal level.

### Spleen lymphocyte proliferation

**Figure 2:**
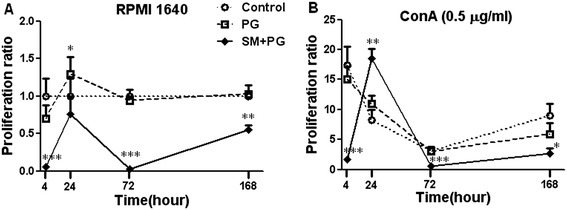
**Dynamic changes of the spleen lymphocyte proliferation in mice exposed to sulfur mustard (SM).** Male KM mice were exposed to saline, propylene glycol (PG) and 20 mg/kg SM by subcutaneous injection. Six animals from each group were sacrificed after SM exposure at 4 h, 24 h, 72 h and 168 h, and the spleens were prepared in a cell suspension as described in the Materials and Methods, with resulting spleen lymphocytes suspended in RPMI 1640 with 10% FBS and Concanavalin A (Con A) (0.5 μg/ml). At 56 h after Con A stimulation, 10 μl ^3^H-TdR was added to each slot. Spleen lymphocyte proliferation was assayed by measuring the ^3^H-TdR of the original proliferation (RPMI 1640) **(A)** and T lymphocytes (Con A) **(B)** described in Section 2. The proliferation ratio of the control group was set to 1.0, and data were presented as the fold increases compared with their respective vehicle controls. *Significant difference between the PG treatment and SM exposure group (*P < 0.05, **P < 0.01, ***P < 0.001). Data were presented as the mean ± SD, *n* = 6 in each group **(A-B)**.

The proliferation of peripheral immune cells is directly related to the state of immune function. Previous reports have shown that SM has no effect on CD3^+^ T cell proliferation in a murine model [12]. In this study, we observed spleen lymphocyte proliferation by ^3^H-TdR incorporation at 4 h, 24 h, 72 h and 168 h after SM exposure. Con A was used to induce T lymphocyte proliferation. As shown in Figure (Figure 2A), 4 h, 24 h, 72 h and 168 h after exposure, lymphocyte proliferation in the SM exposure group was significantly lower than the control group. While Con A-induced T cell proliferation of the SM-exposed group was significantly increased at 72 h and the T cell proliferation levels of the SM-exposed group at 4 h, 24 h, and 168 h were all lower than the PG group (Figure 2B).

### Percentage of CD3^+^CD4^+^ and CD3^+^CD8^+^ T lymphocytes

**Figure 3:**
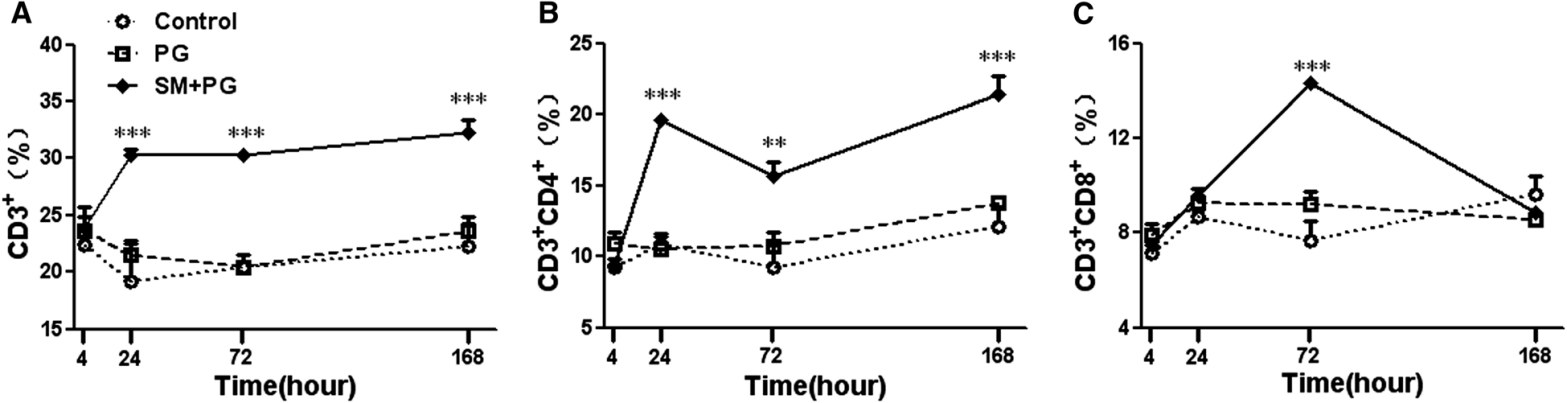
**Dynamic changes of the percentages of CD3** ^**+**^ **, CD3** ^**+**^ **CD4** ^**+**^ **and CD3** ^**+**^ **CD8** ^**+**^ **lymphocytes in mice exposed to sulfur mustard (SM).** Male KM mice were exposed to saline, propylene glycol (PG) and 20 mg/kg SM by subcutaneous injection. Six animals from each group were sacrificed after SM exposure at 4 h, 24 h, 72 h and 168 h, and the spleens were prepared as a cell suspension as described in the Materials and Methods and then incubated with CD3^+^CD4^+^ and CD8^+^ antibodies. The percentages of CD3^+^ **(A)**, CD3^+^CD4^+^ **(B)** and CD3^+^CD8^+^ **(C)** cells were assayed with a flow cytometer (FCM). Data were presented as the mean ± SD, *n* = 6 for each group **(A-C)**. *Significant difference between the PG treatment and SM exposure groups (**P < 0.01, ***P < 0.001).

Previous reports showed that the percentages of CD3^+^CD4^+^ and CD3^+^CD8^+^ T lymphocytes were increased in hairless guinea pigs [15], but the changes in the percentages for mice were still unknown. We found that the CD3^+^ T cells in the spleen were increased gradually after 24 h, 72 h, and 168 h of SM exposure in the SM group (Figure 3A). The percentages of CD3^+^CD4^+^ T cells were significantly higher than the PG group 24 h, 72 h and 168 h after exposure (*P <* 0.01 or 0.001), shown in Figure 3B. The percentage of CD3^+^CD8^+^ T cells was increased at 4 h after SM exposure, and it peaked at 72 h following SM exposure (*P* < 0.001) and then returned to the normal level at 168 h, as shown in Figure 3C.

### Changes in the proinflammatory and anti-inflammatory cytokines

**Figure 4:**
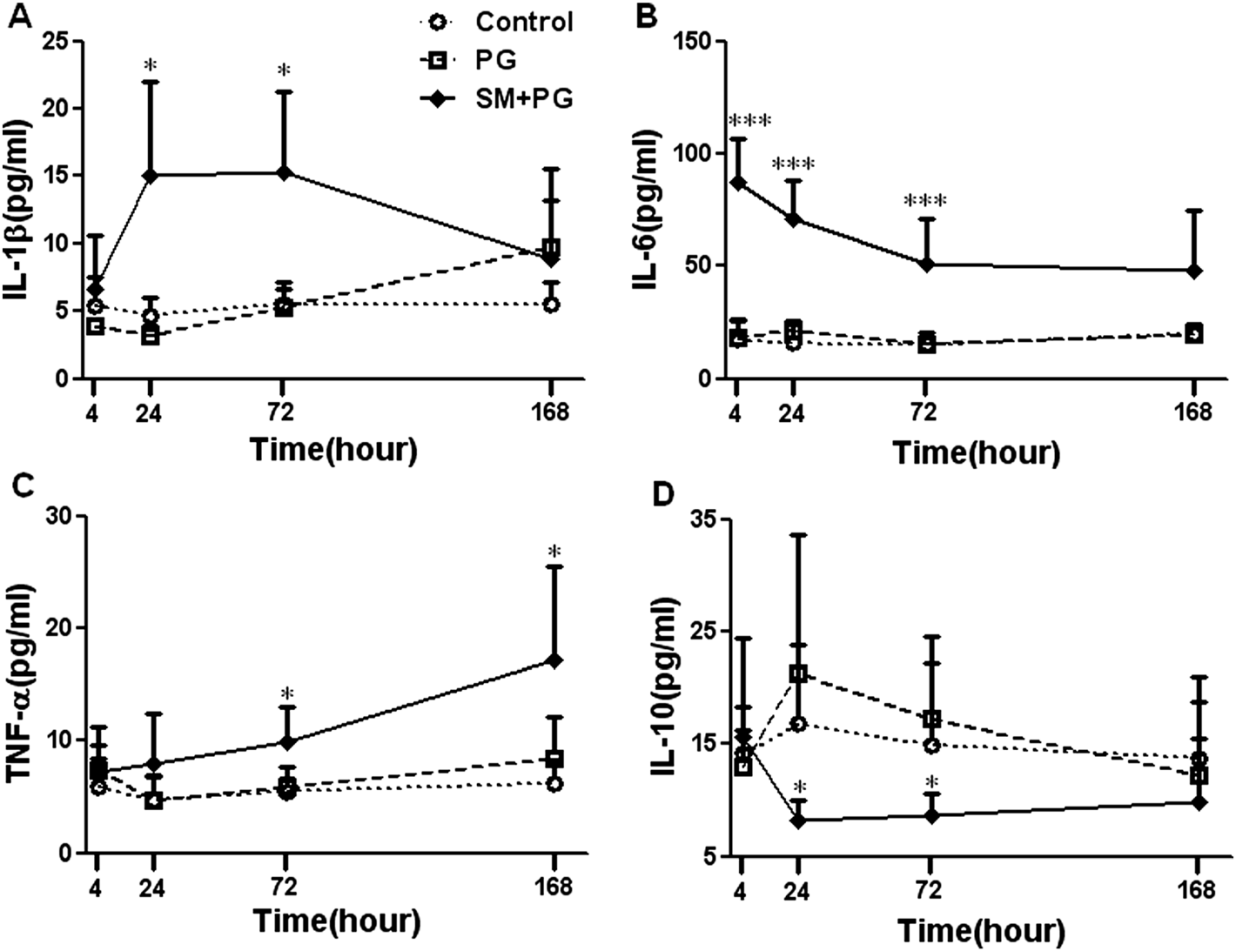
**Dynamic changes in the plasma levels of TNF-α, IL-1β, IL-6 and IL-10 in mice exposed to sulfur mustard (SM).** Male KM mice were exposed to saline, propylene glycol (PG) and 20 mg/kg SM by subcutaneous injection. Six animals from each group were sacrificed after SM exposure at 4 h, 24 h, 72 h and 168 h, blood samples were collected and plasma samples were obtained as described in Materials and Methods. The IL-1β **(A)**, IL-6 **(B)**, IL-10 **(C)** and TNF-α **(D)** levels in plasma were assayed and analyzed by Luminex200. Data are presented as the mean ± SD, *n* = 6 for each group **(A-D)**. *Significant difference between the PG treatment and SM exposure groups (*P < 0.05, **P < 0.01, ***P < 0.001).

To observe the effects of SM on the balance of pro-inflammatory and anti-inflammatory cytokines, the levels of proinflammatory cytokines, IL-1β, IL-6, and TNF-α, and the anti-inflammatory cytokine, IL-10, were observed in this study. In the SM group, the IL-1β levels in the plasma at 24 h and 72 h were significantly higher than the PG group (*P* < 0.05), as shown in Figure 4A. The IL-6 levels were decreased in a time-dependent manner and were significantly higher than the PG group at 4 h, 24 h and 72 h after exposure (*P* < 0.001), as shown in Figure 4B. The level of TNF-α was increased in a time-dependent manner and it was higher than the PG group at 72 h and 168 h after SM exposure (*P* < 0.05), as shown in Figure 4C. The IL-10 level in the SM group was decreased from 4 h to 168 h after SM exposure and lower than the PG group at 24 h and 72 h (*P* < 0.05, Figure 4D).

### Apoptosis and necrosis of lymphocytes

**Figure 5:**
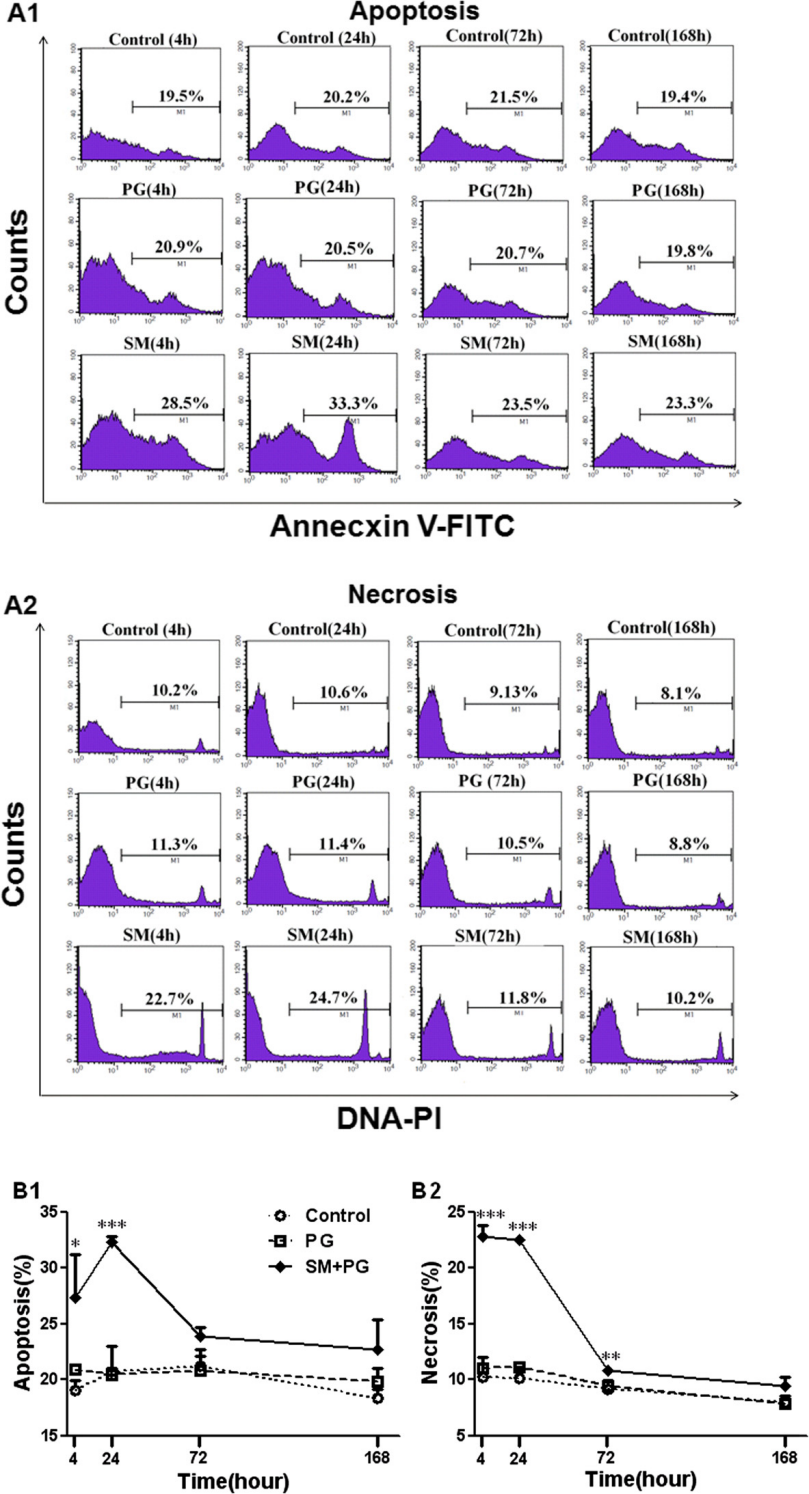
**Apoptosis and necrosis of splenic lymphocytes in mice exposed to sulfur mustard (SM).** Male KM mice were exposed to saline, propylene glycol (PG) and 20 mg/kg SM by subcutaneous injection. Six animals from each group were sacrificed after SM exposure at 4 h, 24 h, 72 h and 168 h, and the spleens were quickly collected. The spleens were prepared as cell suspensions as described in the Materials and Methods and then incubated with FITC and PI. Spleen lymphocyte necrosis (PI^+^) and apoptosis (FITC^+^PI^+^) were assayed with a flow cytometer (FCM) **(A1-A2)**. Data are presented as the mean ± SD, *n* = 6 for each group **(B1-B2)**. *Significant difference between the PG treatment and SM exposure groups (*P < 0.05, **P < 0.01, ***P < 0.001).

The spleen lymphocyte apoptosis and necrosis percentages in the SM group were significantly increased. The spleen lymphocyte apoptosis percentage was higher than the PG group at 4 h and 24 h (*P* < 0.05 and *P* < 0.001), as shown in Figure 5A1 and B1. Compared to the PG group, the necrosis of the SM group was significantly higher than the PG group at 4 h, 24 h and 72 h (*P <* 0.01 or *P <* 0.001), as shown in Figure 5A2 and B2.

### DNA damage of the bone marrow cells

**Figure 6:**
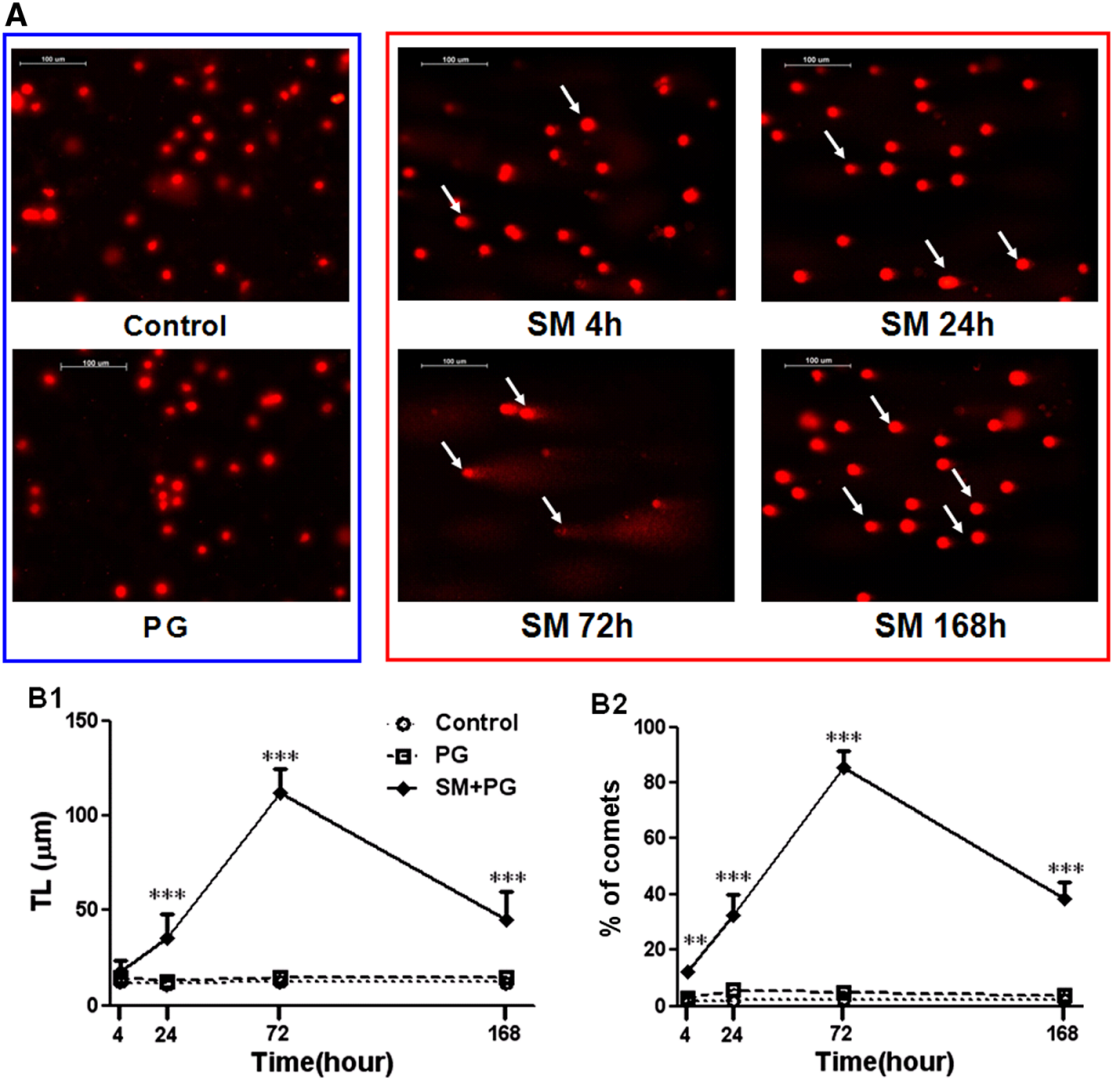
**DNA damage of the bone marrow cells in mice exposed to sulfur mustard (SM).** Male KM mice were exposed to saline, propylene glycol (PG) and 20 mg/kg SM by subcutaneous injection. Six animals from each group were sacrificed after SM exposure at 4 h, 24 h, 72 h and 168 h, and a bone marrow cell suspension was prepared and assayed by single cell gel electrophoresis technique (SCGE) as described in the Materials and Methods **(A)**. The TL **(B1)** and the percentage of comets **(B2)** were evaluated to assess SM-induced DNA damage. Data are presented as the mean ± SD, *n* = 6 for each group **(B1-B2)**. *Significant difference between the PG treatment and SM exposure groups (**P < 0.01, ***P < 0.001).

As an alkylating agent, SM is particularly toxic to rapidly proliferating cells, such as lymphoid and bone marrow cells. It was reported that bone marrow T or stem cells have an important immunoregulation function [22,23]. Bone marrow cell proliferation and differentiation were so active that DNA was sensitive to SM alkylation [24,25]. Therefore, we observed DNA damage of the bone marrow nucleated cells after SM exposure by SCGE. The bone marrow cells appear to demonstrate remarkably dynamic DNA damage after SM exposure (Figure 6A). The percentage of commets and the "tail length" (TL) had positive correlation with the degree of DNA damage. At 4 h after exposure, approximately 12% of the cells were damaged in the SM group, but the TL was not significantly different from the PG group. At 24 h after exposure, the percentage of comets reached 30%, and the TL was three times longer than that of the PG group. At 72 h, the percentage of comets reached 85%, and the TL was 10 times that of the control group, and the number of cells was greatly reduced. At 168 h after exposure, the percentage of comets, TL and number of bone marrow cells in the SM group were decreased, but their numbers were still significantly different compared to the PG group (Figure 6B).

## Discussion

SM is a powerful chemical warfare agent (CWA) that was extensively used during World War I/II and more recently in the Iran-Iraq war of the 1980s. In addition to its military use, it is a potential weapon of mass destruction against civilians that causes both acute and chronic health effects [26,27]. The chemical weapons discarded by invading Japanese troops during the War of Resistance against Japanese Invasion are threatening the safety and homes of Chinese people. Forty-three people were poisoned by abandoned Japanese mustard gas, which left one person dead in August 2003. Several researchers found that peripheral blood T lymphocytes, monocytes, macrophages, neutrophils, endothelial and epithelial cells in the gut as well as bone marrow cells were sensitive to SM. These cell types were susceptible to apoptosis and necrosis, and the main reason for necrosis may be related to DNA breaks by SM alkylation [28,29]. Other important injury factors, such as inflammatory and oxidative stress, may be important cofactors in increasing the toxicity of SM [30].

The effect of SM on human T lymphocyte proliferation has not been previously reported. Some animal studies have reported that SM exposure causes severe lymphocyte function suppression with progressively decreased splenocyte proliferation that recovered up to the fifth day after exposure [31]. While others have reported that SM does not depress murine T lymphocyte proliferation in response to Concanavalin A or to an anti-CD3 antibody, B cell proliferation in the presence of lipopolysaccharide is not significantly impaired [12]. In this study, we found that, in a mouse model induced by subcutaneous injection, the primary proliferations of splenocytes at 4, 24, 72 and 168 h were significantly lower than in the PG group, and the Con A-induced T cell proliferation was significantly lower than in the PG group at 4 h, 72 h and 168 h, while 24 h was significantly higher. Coutelier *et al*. [12] found that SM-induced systemic intoxication did not depress T lymphocyte function as their proliferation in response to concanavalin A or to an anti-CD3 antibody was not affected by the treatment. Another study showed that cyclophosphamide, a commonly used chemotherapy drug and alkylating agent, could adjust immune function by enhancing lymphocyte proliferation in animal models [32]. SM is an alkylating agent with a similar mechanism of action to that of cyclophosphamide. Therefore, the reason that a dose lower than the LD50 of SM exposure caused T and B cell proliferation rebound at an early time of SM intoxication may be similar to the enhancement of cyclophosphamide to immune cell proliferation as a hormesis.

Twenty years after SM exposure, victims of the Iran-Iraq war had a lower CD4^+^ level and higher CD8^+^ level in plasma compared to control subjects, suggesting that the SM caused a long-term suppression of the immune system [13]. Our results in a mouse model found that the spleen weights were significantly decreased at 24 h and 72 h, and they gradually recovered to a control level at 168 h after a high-dose (20 mg/kg) of SM. Spleen lymphocyte apoptosis and necrosis were observed at 4 h and recovered after 72 h. In T cell subsets, the percentages of CD3^+^CD4^+^ and CD3^+^CD8^+^ T cells were higher than the PG group. The percentage of CD3^+^CD4^+^ T cells was higher than the PG group, which may be due to the decrease in the number of mononuclear macrophages, which was reduced 90% in human blood [11]. These results are consistent with other studies reporting observations after percutaneous exposure and inhalation of SM in which researchers not only observed an increasing percentage of CD4^+^ and CD8^+^ T cells in tissues and body fluids, which is consistent with our results. These studies also observed an increasing ratio of CD4^+^/CD8^+^ T cells and inflammatory injuries at exposure positions, suggesting that SM systemic exposure selectively affects T cell activity and the percentage of T cell subsets as well as causes an imbalance of related inflammatory cytokines [15].

Many studies on immunological and inflammatory changes in skin and corneas after acute intoxication as well as long-term chronic immune system diseases caused by SM exposure have been reported [15,33-36]. Many cytokines, such as TNF-α, IL-1α, IL-1β, IL-4, IL-6, IL-8, IL-10, IL-12, IL-13, IL-15, IL-17, TGF-β, G-CSF, IGF-1 (insulin-like growth factor-1), EGF (epidermal growth factor), GM-CSF, and IFN-γ, are closely related to the toxicity of SM [15,37-40]. The levels of these cytokines vary in different SM injuries. For example, IL-17 likely plays an important role in the pathogenesis of SM-induced lung injury [39]. The numbers and proliferation activity of T cells could affect the levels of many cytokine types [39,41,42]. A variety of cytokines secreted by T cells, including IL-1β, IL-6, IL-10 and TNF-α, are reported to be associated with respiratory damage. IL-1β, IL-6 and TNF-α are pro-inflammatory cytokines [43-45], while IL-10 is closely related to the anti-inflammatory mechanisms of the body [46]. In this research, we observed dynamic changes in the pro-inflammatory cytokines (TNF-α, IL-1β and IL-6) and anti-inflammatory cytokine (IL-10). We found that the IL-1β levels in plasma were significantly higher than the PG group at 24 h and 72 h after SM exposure, but the TNF-α level did not increase significantly until 72 h after exposure. Some studies demonstrated that the IL-1β and TNF-α levels were significantly increased in HaCaT cells and respiratory alveolar exudates, which was associated with skin irritation and blistering airway inflammatory exudate 6–48 h after SM exposure [40,47]. The time points of increasing the IL-1β and TNF-α levels were different from our results, which may be related to the various tissue distributions from various exposure models. We found that each of the two inflammatory cytokines induced injury at different time points and led to an inflammatory-sensitive state with an increase in IL-1β at the early phase and in TNF-α at the late phase. As a proinflammatory cytokine [43], the level of IL-6 was increased after SM exposure in our study. Then, when splenocyte apoptosis increased and necrosis reduced, the IL-6 level gradually decreased from a significantly higher level, but it was still significantly higher than the PG group at all four time points. Interestingly, when the IL-6 level was highest at 4 h, the splenocyte apoptosis percentage of the SM group was not significantly different compared to the PG group; meanwhile, the necrosis percentage was also at the highest level. *In vivo* studies found that the serum IL-6 was increased in patients with sulfur mustard poisoning and COPD [48]. *In vitro*, treatment with 100 μmol/L of SM for 24 hours resulted in a significantly increased level of IL-6 secretion by human epidermal keratinocytes (HEKs) and human skin fibroblasts (HSFs) [49]. These results were consistent with our finding that the IL-6 level increased after SM exposure. The main sources of IL-6 are monocytes, macrophages, T and B cells, fibroblasts, epithelial cells, and smooth muscle cells [48]. Therefore, we speculated that the gradual decrease in IL-6 was mainly due to the significant necrosis and apoptosis of T and B lymphocytes in the intoxicated spleen. IL-10 is an anti-inflammatory cytokine [50,51] that is produced by various types of activated immune cells. The IL-10 levels were significantly lower than the PG group at 24 h and 72 h, while the spleen coefficient, T-lymphocyte proliferation and apoptosis proportion were all at the lowest level. The decrease in IL-10 suggested a low anti-inflammatory capacity, which might relate to the general toxic injuries. These results demonstrated that SM exposure could cause an imbalance of the pro-inflammatory and anti-inflammatory cytokines.

The immune cells (especially T and B cells) are originated from bone marrow cells [22,23,52,53], which is one of the most sensitive tissues to SM alkylation due to its hyperactive proliferation and differentiation. DNA damage is reported as a key consequence of SM exposure, occurring either via direct DNA alkylation by SM or via oxidative or nitrosative stress [54,55]. Therefore, we observed dynamic DNA damage of the bone marrow cells in the SM subcutaneous injection mouse model with SCGE. DNA damage in the bone marrow cells was apparent at 4 h and increased to peak at 72 h; it then decreased at 168 h, but it was still higher than the PG group. The result was similar to the dynamic changes of apoptosis and necrosis in splenocytes, indicating that DNA damage may be the main and direct reason of cell apoptosis and necrosis, and the difference in the time course may be due to the differential tissue distribution of SM [56].

## Conclusions

SM significantly inhibited T lymphocyte proliferation and increased the percentages of CD3^+^CD4^+^ and CD3^+^CD8^+^. This may be due to the SM-induced apoptosis and necrosis of T lymphocytes, the disturbance of proinflammatory and anti-inflammatory cytokines, and DNA damage in bone marrow cells. These results improve our understanding of immune function in an SM toxicity mouse model.

## Abbreviations

Con A: Concanavalin A
CPM: Counters per minute
CWA: Chemical warfare agent
EGF: Epidermal growth factor
FCM: Flow cytometer
GM-CSF: Granulocyte-macrophage colony stimulating factor
HEKs: Human epidermal keratinocytes
HSFs: Human skin fibroblasts
IACUC: Institutional Animal Care and Use Committee
IGF: Insulin-like growth factor
IL: Interleukin
PG: Propylene glycol
PI: Propidium iodide
SCGE: Single cell gel electrophoresis technique
SM: Sulfur mustard
TL: Tail length
TNF: Tumor necrosis factor
